# Patient-reported lower urinary tract symptoms after hysterectomy or hysteroscopy: a study from the Swedish Quality Register for Gynecological Surgery

**DOI:** 10.1007/s00192-017-3268-9

**Published:** 2017-01-23

**Authors:** Mathias Pålsson, Jan-Henrik Stjerndahl, Gabriel Granåsen, Mats Löfgren, Karin Sundfeldt

**Affiliations:** 1 Department of Obstetrics and Gynaecology, Sahlgrenska Academy at Gothenburg University, Gothenburg, Sweden; 2 Department of Obstetrics and Gynaecology, Sahlgrenska University Hospital, SE-413 45 Gothenburg, Sweden; 3 Department of Public Health and Clinical Medicine, Epidemiology and Global Health, Umeå University, Umeå, Sweden; 4 Department of Clinical Science, Obstetrics and Gynaecology, Umeå University Hospital, Umeå, Sweden

**Keywords:** Hysterectomy, Hysteroscopy, Quality register, Urinary incontinence, Urinary urgency

## Abstract

**Introduction and hypothesis:**

Hysterectomy is sometimes considered the cause of lower urinary tract symptoms (LUTS). We hypothesized that hysterectomy for abnormal uterine bleeding and/or symptoms of fibroids is more likely to cause LUTS than a hysteroscopic procedure for the same indications.

**Methods:**

Two groups of women were compared: one group comprised 3,618 women who had had a hysterectomy due to abnormal uterine bleeding or symptoms of fibroids and the other group comprised 238 women who had had hysteroscopic treatment for the same indications. The main outcome measures were occurrence of LUTS before and 1 year after the surgical intervention. The frequencies of LUTS before and after surgery were compared between the groups. Binary logistic regression was used to model the odds of having postoperative urinary leakage and urgency while controlling for uterine size, surgical procedure and preoperative LUTS.

**Results:**

There were no statistically significant differences between women after hysterectomy and after hysteroscopy in the frequencies of LUTS before or after surgery, when uterine size was comparable. However, there was a difference in the rates of de novo urinary incontinence between women with hysterectomy and women with hysteroscopy (7.6%, 95% CI 6.3–9.0, and 3.2%, 95% CI 1.6–6.5, respectively). Of the women with a large uterus, 58.6% (95% CI 51.5–65.5) reported relief of urinary incontinence and 85.5% (95% CI 82.3–88.4) reported relief of urinary urgency postoperatively.

**Conclusions:**

Our results suggest that it is important to individualize preoperative information in women prior to hysterectomy since the outcome concerning LUTS depends on preoperative symptoms and uterine size.

## Introduction

Hysterectomy is one of the most common major gynaecological procedures. In some countries there is a trend towards a decrease in hysterectomy rates [1–3], but it is still a common procedure. Hysterectomy is an effective way to treat several benign disorders [4] and is often necessary for the treatment of malignant gynaecological conditions. Studies have shown high levels of satisfaction postoperatively [5] and it is considered to be a relatively safe procedure with major complications in approximately 4% of patients [6] and mortality rates in the range 0.2–1 in 1,000 hysterectomies [7, 8]. Lower urinary tract symptoms (LUTS), e.g. urinary leakage, urgency and nocturia, are a common problem in women. The prevalence of LUTS is reported to range from 25% to 45% [3, 9] and negatively affects quality of life [10]. In Sweden, LUTS has been reported to be responsible for approximately 2% of the total health-care costs [11].

There has been and still is a lively discussion amongst gynaecologists about whether hysterectomy enhances the risk of developing LUTS [12–17]. Surgical trauma and/or altered anatomy are considered to be the causes [18]. Treatments other than hysterectomy are becoming more common for some benign gynaecological conditions such as abnormal uterine bleeding or fibroids, and have shown good efficacy [19, 20]. Therefore, it has become even more important to assess the results of hysterectomy and its potential side effects.

Cross-sectional and retrospective studies have tended to show an increased risk of incontinence after hysterectomy [13, 14]. In contrast, prospective studies in which preoperative data are available tend to show the opposite: that hysterectomy does not increase the risk of urinary incontinence [15, 17]. Cross-sectional and retrospective studies usually lack information as to whether the patients had urinary incontinence prior to hysterectomy or rely on patient recall for the information [13, 14, 18]. Prospective studies are more likely to include information regarding preoperative incontinence [15–17].

In this study we evaluated the outcomes regarding urinary incontinence and urgency after a hysterectomy with a focus on both de novo symptoms and problems that existed preoperatively. The study population comprised women with abnormal uterine bleeding and/or symptoms of fibroids who had either a hysterectomy or a hysteroscopic procedure with endometrial resection or ablation or removal of submucosal fibroids. Those who underwent a hysteroscopic procedure served as the control group, since the same conditions are treated without removal of the uterus, and potential damage to the surrounding tissues and alteration of the anatomy in the pelvis are avoided. The choice between procedures often depends on the gynaecologist’s preference rather than the patient’s characteristics, except in relation to certain parameters such as uterine size and location of fibroids.

## Materials and methods

This study used data collected through the Swedish National Quality Register for Gynaecological Surgery (GynOp register) that was established in 1997. From the beginning preoperative, perioperative and postoperative information in women undergoing endometrial ablation or hysterectomy has been gathered. Gradually the GynOp register has expanded and today encompasses all major surgical fields in gynaecology. The GynOp register covers approximately 70% of the Swedish female population. Information about the register is available from the GynOp website (http://www.gynop.org/english/english.htm). Data are collected prospectively and consist of both patient questionnaires and forms completed by the gynaecologist. Data concerning general health history as well as current symptoms are collected by questionnaires before and after surgery. Patients’ willingness to participate has been evaluated and the results show that the questionnaires are well accepted and the response rate is over 90% [21]. The gynaecological surgeon records data on clinical status, surgical procedure and postoperative care.

In this study the patients’ answers to questions regarding LUTS were combined with data on the procedure they had undergone. Data used in the present study were collected from the start in 1997 to 2006 when there was a major revision of the GynOp register. During this time period basic questions regarding LUTS included “Do you have either/both of the following bothers from the lower abdomen?”: urinary incontinence/urinary leakage (yes or no), or urinary urgency (yes or no). The questions were subject to face and content validation. The questions were not designed to measure LUTS objectively, only to determine whether respondents were bothered by incontinence or urgency. During the study period the procedure was changed from collecting long-term outcome of the surgical procedure from the patients at 6 months and 2 years postoperatively to one questionnaire at 1 year postoperatively.

**Fig. 1:**
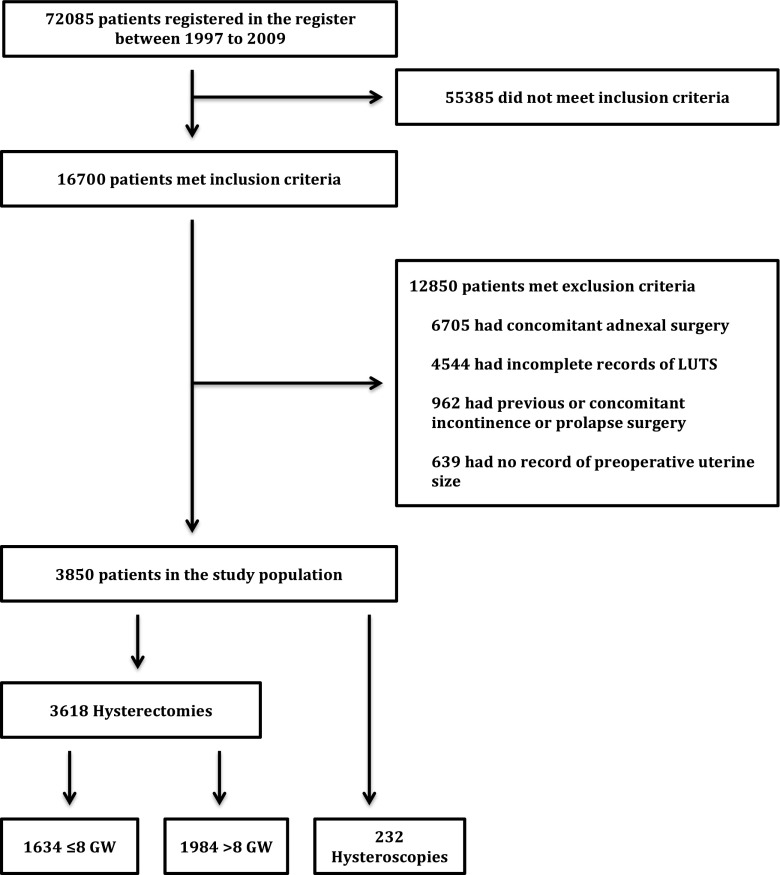
Flow chart of the inclusion and exclusion process (*LUTS* lower urinary tract symptoms, *GW* gestational weeks)

The process of exclusion and inclusion of women who underwent a hysterectomy or hysteroscopy between 1997 and 2006 is shown as a flow chart in Fig. 1. The data were collected in 2009 and included data from the original questionnaires only.

Inclusion criteria were hysterectomy or hysteroscopy due to abnormal uterine bleeding and/or symptoms of uterine fibroids and age 40–52 years and uterine size recorded by the surgeon. In the GynOp register during the study period, abnormal uterine bleeding or fibroids treated with hysteroscopy were seldom found in women older than 52 years. Polyps or postmenopausal bleeding were then a more common reason for hysteroscopy. Also, a hysterectomy before 40 years of age was seldom found in the register for the chosen indications. The surgical indications for hysterectomy in patients older than 52 years included mainly malignant (or suspected malignant) conditions and prolapse. We also wanted to avoid too wide a time span, since age itself is considered to be associated with LUTS.

Exclusion criteria were any previous or concomitant prolapse or incontinence surgery to minimize the impact of a poorly functioning pelvic floor, hysterectomy due to malignant (or suspected malignant) conditions, concomitant adnexal surgery to eliminate the risk of iatrogenic menopause, women with an American Society of Anesthesiologists (ASA) physical status classification of III or worse [22], and those who had not fully answered the questions regarding LUTS.

For the statistical analysis the hysterectomy group was divided into two subgroups, one comprising women with a mean uterine size the same as the mean uterine size of the control group (hysteroscopy) and the other comprising women with a larger uterus. There was a substantial difference in the distribution of uterine sizes between the hysterectomy group and the hysteroscopy control group. The heavily skewed uterine size data prevented matching the uterine sizes in the control group to all uterine sizes in the hysterectomy group and made the use of multivariate analysis inappropriate for determining whether uterine size or mode of surgery had any impact on LUTS.

### Statistical methods

The chi-squared test was used to test whether the distribution of the answers on each factor were equal in the different diagnostic groups. The *t* test was used to test whether the mean age, mean body mass index (BMI), and mean number of births were equal in the different diagnostic groups. A significance level of 0.05 was used for all tests. All *p* values presented are uncorrected. To adjust for between-group comparisons, the Holm-Bonferroni method was used. If the test was significant after correction, then this is noted in the tables. Binary logistic regression was used to model the odds of having postoperative urinary leakage and urgency while controlling for uterine size, surgical procedure and preoperative LUTS. Receiver operating characteristic curves were used to estimate a threshold for uterine size. SPSS version 22 (IBM Corp., Armonk, NY) and R 3.2.2 (R Core Team, Vienna, Austria) were used for the analyses.

## Results

After the inclusion and exclusion process the final cohort comprised 3,850 women (Fig. 1). The majority of women (80%) answered their questionnaires 1–2 years after surgery: 786 (20%) at 6 months, 1,642 (41.8%) at 1 year , and 1,510 (38.2%%)at 2 years. Of the 3,856 women. 3,618 had had a hysterectomy and 238 a hysteroscopic procedure. As described above, the hysterectomy group was divided into two subgroups so a valid comparison with the hysteroscopy group was possible. The cut-off value was set according to the mean uterine size. In the GynOp register, uterine size is estimated in a gynaecological examination prior to the surgical intervention by palpation of the uterus, and is reported in terms of gestational weeks (GW) as small, normal, or 6, 8, 10, 12 or 14 GW, etc. A normal-sized uterus is considered a uterus at 4 GW. Thus the women were classified into three groups: group 1 comprised those with hysterectomy and ≤8 GW, group 2 those with hysterectomy and >8 GW, and group 3 all those with hysteroscopy. The mean uterine sizes of groups 1, 2 and 3 were 5.3 GW (95% CI 5.2–5.4 GW), 13.3 GW (95% CI 13.2–13.5 GW) and 5.3 GW (95% CI 5.0–5.6 GW), respectively. This classification is somewhat arbitrary with regard to LUTS symptoms since there is probably no difference clinically between uterine sizes 8 GW and 9 GW.

We also tested classifiers other than the mean uterine size. A receiver operating characteristic curve was created to determine a possible uterine size threshold for predicting LUTS. The best accuracy (area under the curve 0.52) was noted for 9.5 GW, indicating that uterine size is a poor prognostic factor for postoperative LUTS.

The basic preoperative characteristics of the women are shown in Table 1. Uterine size, age, BMI, and parity were similar in group 1 and group 3. Uterine size was significantly larger in group 2 than in groups 1 and 3. Group 2 had had fewer gynaecological surgery procedures than groups 1 and 3. Further analysis revealed that women in group 2 had had fewer tubal ligations and lesser cervical surgery (dysplasia) than women in groups 1 and 3.

**Table 1:** Characteristics of the women according to uterine size group and mode of surgery

Characteristic	Hysterectomy		Hysteroscopy	*p* values		
	Group 1 (uterine size ≤8 GW, *n* = 1,634)	Group 2 (uterine size >8 GW, *n* = 1,984)	Group 3 (*n* = 238)	Group 1 vs. 3	Group 2 vs. 3	Group 1 vs. 2
Age (years), mean (SD)	45.1 (3.0)	46.0 (3.1)	44.9 (3.1)	0.397	<0.001*	<0.001*
BMI (kg/m^2^), mean (SD)	25.7 (4.6)	25.3 (4.1)	26.2 (4.6)	0.102	0.006*	0.023*
Parity, mean (SD)	2.4 (1.1)	2.1 (1.1)	2.4 (1.1)	0.689	<0.001*	<0.001*
Uterus size (gestational weeks), mean (SD)	5.3 (1.8)	13.3 (3.4)	5.3 (2.2)	0.689	<0.001*	<0.001*
ASA classification 1, % (95% CI)	91.1 (89.6–92.4)	93.1 (91.8–94.2)	88.6 (83.8–92.1)	0.221	0.014*	0.031
Hypertension, % (95% CI)	15.7 (14.0–17.6)	13.2 (11.8–14.8)	18.1 (13.7–23.4)	0.358	0.039	0.031
Smokers, % (95% CI)	28.9 (26.8–31.2)	21.4 (19.6–23.2)	20.2 (15.6–25.7)	0.005*	0.668	<0.001*
Health issues other than gynaecological, % (95% CI)	64.5 (62.1–66.8)	58.6 (56.4–60.8)	60.8 (54.4–66.8)	0.267	0.521	<0.001*
Previous gynaecological surgery, % (95% CI)	80.3 (78.3–82.2)	66.0 (63.9–68.1)	81.5 (76.1–85.9)	0.661	<0.001*	<0.001*

*GW* gestational weeks, *CI* confidence interval

*Considered statistically significant after Holm-Bonferroni correction for multiple testing.

The data regarding urinary symptoms are presented in Table 2. There were no differences between groups 1 and 3. Of women with a larger uterus (group 2), 8.0% reported urinary leakage after hysterectomy, and of those with a smaller uterus (group 1), 11.8% reported leakage (*p* < 0.001). Of women in group 2, 27.9% reported urgency before surgery compared with 17.7% in group 1 and 11.2% in group 3 (*p* < 0.001 in both cases). One year after surgery this result was reversed.: only 6.7% of women in group 2 reported urgency (a fourfold decrease), a rate lower than in group 1 (11.4%, *p* < 0.001) and group 3 (11.6% *p* = 0.004).

**Table 2:** Urinary symptoms before and after surgery

	Hysterectomy				Hysteroscopy		*p* values		
	Group 1 (uterine size ≤8 GW, *n* = 1,634)		Group 2 (uterine size >8 GW, *n* = 1,984)		Group 3 (*n* = 238)		Group 1 vs. 3	Group 2 vs. 3	Group 1 vs. 2
	*n*	% (95% CI)	*n*	% (95% CI)	*n*	% (95% CI)			
Urinary incontinence									
Before surgery	157	9.6 (8.3–11.1)	203	10.2 (9.0–11.6)	21	8.8 (5.8–13.1)	0.789	0.570	0.570
After surgery	193	11.8 (10.3–13.5)	158	8.0 (6.9–9.2)	23	9.7 (6.5–14.1)	0.390	0.435	<0.001*
Urinary urgency									
Before surgery	290	17.7 (16.0–19.7)	553	27.9 (25.9–29.9)	31	13.0 (9.3–17.9)	0.087	<0.001*	<0.001*
After surgery	186	11.4 (9.9–13.0)	133	6.7 (5.7–7.9)	29	12.2 (8.3–15.2)	0.800	0.003*	<0.001*

*GW* gestational weeks, *CI* confidence interval

*Considered statistically significant after Holm-Bonferroni correction for multiple testing.

**Fig. 2:**
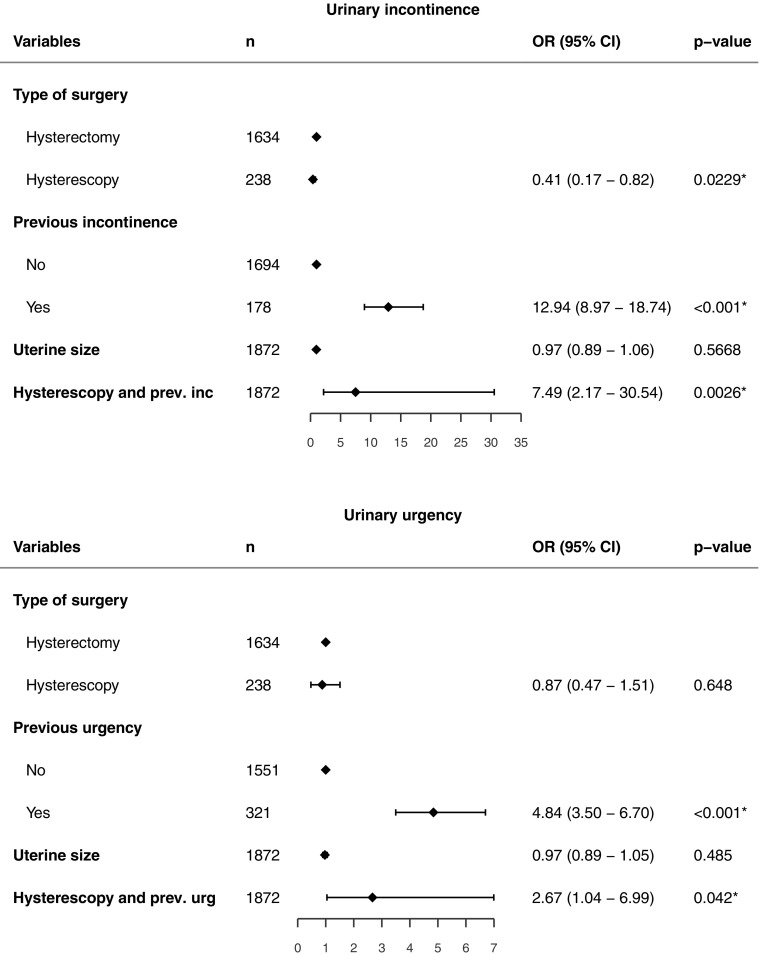
Binary logistic regression analysis of the occurrence of postoperative urinary incontinence and urgency in groups 1 and 3. Hysteroscopy and previous incontinence and urgency are additionally associated with preoperative LUTS and a hysteroscopy. *p < 0.05

In the multiple regression analysis uterine size was not associated with the postoperative occurrence of LUTS in groups 1 and 3 (odds ratio, OR, 0.97, 95% CI 0.89–1.06, *p* = 0.567, for incontinence; OR 0.97, 95% CI 0.89–1.05, *p* = 0.485, for urgency; Fig. 2). In group 2 (>8 GW), an increase in uterine size of 2 GW had a slight protective effect against LUTS (OR 0.93, 95% CI 0.88–0.98, *p* = 0.011, for incontinence; OR 0.94, 95% CI 0.88–0.99, *p* = 0.03), for urgency).

The results of analysis of outcomes regarding LUTS according to urinary symptoms reported before surgery are shown in Table 3. There was no difference between groups 1 and 3 with regard to the presence of urinary incontinence before surgery. After surgery, the proportion of women in group 1 reporting de novo urinary incontinence was higher than in group 3 (7.6% vs. 3.2%, *p* = 0.015). This was also found in the multivariate analysis, which showed that hysteroscopy reduced the risk of de novo incontinence (OR 0.41, 95% CI 0.17–0.82, *p* = 0.023) more than hysterectomy (Fig. 2). Of women with a small uterus (group 1), 7.6% reported de novo urinary incontinence, a significantly higher proportion than among women with a large uterus (group 2, 4.2%, *p* < 0.001). If incontinence was present before surgery, the risk of incontinence was significantly higher after hysteroscopy than after hysterectomy (OR 7.49, 95% CI 2.17–30.54, *p* = 0.0026). Moreover, a high proportion of women (58.6%) with a uterus >8 GW reported relief of urinary leakage 1 year after surgery.

**Table 3:** Occurrence of urinary incontinence after surgery in relation to status before surgery

	Hysterectomy				Hysteroscopy		*p* values		
	Group 1 (uterine size ≤8 GW (*n* = 1,634)		Group 2 (uterine size >8 GW(*n* = 1,984)		Group 3 (*n* = 238)		Group 1 vs. 3	Group 2 vs. 3	Group 1 vs. 2
	*n*	% (95% CI)	*n*	% (95% CI)	*n*	% (95% CI)			
Patients without urinary incontinence before surgery									
Continent before surgery	1,477	100	1,781	100	217	100			
Incontinent after surgery	112	7.6 (6.3–9.0)	74	4.2 (3.3–5.2)	7	3.2 (1.6–6.5)	0.028	0.636	<0.001*
Patients with urinary incontinence before surgery									
Incontinent before surgery	157	100	203	100	21	100			
Incontinent after surgery	81	51.6 (43.8–59.6)	84	41.4 (34.8–48.3)	16	76.2 (54.9–89.4)	0.058	0.005*	0.068

*GW* gestational weeks, *CI* confidence interval

*Considered statistically significant after Holm-Bonferroni correction for multiple testing.

Analysis of urgency revealed no difference between groups 1 and 3 in the frequency of de novo urgency 1 year after surgery (Table 4). The risk of still having bothersome urgency after surgery was higher after hysteroscopy than after hysterectomy (OR 2.67, 95% CI 1.04–6.99, *p* = 0.042). Group 2 had a lower rate of de novo urgency than groups 1 and 3 (*p* < 0.001 and *p* = 0.015). Among women with symptoms present before surgery, 28.6% of those in group 1 had a significant decrease in urgency postoperatively (95%CI 23.7–34.1) compared with 48.4% of those in group 3 (95% CI 32.0–65.2, *p* = 0.038). An even larger proportion of women in group 2 had a decrease in urgency after surgery: 85.5% reported relief of urgency (*p* < 0.001 compared with both groups 1 and 3).

**Table 4:** Occurrence of urinary urgency after surgery in relation to status before surgery

	Hysterectomy				Hysteroscopy		*p* values		
	Group 1 (uterine size ≤8 GW (*n* = 1,634)		Group 2 (uterine size >8 GW (*n* = 1,984)		Group 3 (*n* = 238)		Group 1 vs. 3	Group 2 vs. 3	Group 1 vs. 2
	*n*	% (95% CI)	*n*	% (95% CI)	*n*	% (95% CI)	1 vs 3	2 vs 3	1 vs 2
Patients without urinary urgency before surgery									
No urgency before surgery	1,344	100	1,431	100	207	100			
Urgency after surgery	103	7.7 (6.4–9.2)	53	3.7 (2.8–4.8)	14	6.8 (4.1–11.0)	0.753	0.059	<0.001*
Patients with urinary urgency before surgery									
Urgency before surgery	290	100	553	100	31	100			
Urgency after surgery	83	28.6 (23.7–34.1)	80	14.5 (11.8–17.6)	15	48.4 (32.0–65.2)	0.039*	<0.001*	<0.001*

*GW* gestational weeks, *CI* confidence interval

*Considered statistically significant after Holm-Bonferroni correction for multiple testing.

The results were further evaluated with regard to the mode of hysterectomy in the total cohort and within each group. There were no significant differences in the frequencies of urinary symptoms before and after hysterectomy among those who underwent abdominal, laparoscopic and vaginal hysterectomy (data not shown). In the patients in this study undergoing hysterectomy, by far the greatest proportion underwent abdominal hysterectomy (67.6% of all procedures); vaginal hysterectomy accounted for 25.8% and laparoscopic only 6.6% of procedures. Vaginal hysterectomy was more common in group 1 (42.2%).

## Discussion

### Main findings

Several epidemiological studies have shown an increased risk of urinary incontinence after hysterectomy [13, 14, 18]. Our results, which were based on prospective data from a large population of patients registered with GynOp, suggest the need for risk stratification of patients depending on whether LUTS is present before surgery. In addition, uterine size was found to affect outcome. Of women with urinary urgency preoperatively and a uterus with a size >8 GW, 85.5% stated at follow-up that the urgency symptoms had disappeared. This is in line with the findings of several prospective studies in women with known preoperative status regarding LUTS. One study showed a decrease in stress incontinence after surgery from 29.5% to 10% over a 2-year follow-up [15]. Another study showed a decrease in stress urinary incontinence from 44% to 31% over a 3-year follow-up [17]. A randomized controlled study comparing outcomes between partial and total hysterectomy showed decreases in self-reported urge incontinence after surgery from 19% to 11% and 16% to 12%, respectively [23]. In our study, only 3.7% of women with a larger uterus reported de novo urgency 1 year after hysterectomy. A significantly lower proportion of these women had de novo urinary incontinence compared with women with a uterine size ≤ 8 GW.

There was a difference in the frequency of LUTS between women with ≤8 GW and those who underwent hysteroscopy (groups with similar uterine sizes). Notably, the proportion of women with de novo urinary incontinence was higher after a hysterectomy, but if LUTS was present before surgery the proportion of women with symptom relief was also higher.

### Strengths and weaknesses

The sample size (3,938 women) is an obvious strength of this study. We were also able to select a homogeneous population because of the large number of registered operations in the GynOp register. The homogeneity reduced the impact of confounding factors such as age, parity and BMI. All data concerning urinary incontinence and urgency were prospectively collected and self-reported preoperatively and postoperatively. This is a great advantage compared to cross-sectional studies that suffer from lack of reliable information regarding preoperative data on LUTS [13, 18, 24]. The mode of data collection also minimized the risk of recall bias, which is a problem in retrospective studies. Taking uterine size into account is important since LUTS has been shown to be less likely in women with a small uterus [25]. Comparing different surgical methods for treating the same disorders in women with equal uterine size makes it possible to rule out the underlying condition as a confounder of the results [26]. We were also able to exclude all women who had concomitant or previous surgery for prolapse or incontinence. As a result, we reduced the impact of already known poor pelvic floor function on the results.

Our reliance solely on patient-reported outcomes and no objective measurements of LUTS could be a limitation. However, other studies have shown a good correlation between patients’ subjective assessment of their continence and objective measurements [27]. In the present study, 26.0% of the women stated preoperatively that they had LUTS. This frequency is in line with the findings of several other studies [3, 9], and hence we regard the questionnaires in the GynOp register as useful for following changes in symptoms after surgery. It is reasonable to believe that each woman described her LUTS symptoms in the same way before and after surgery.

Our follow-up time was short compared to those in several cross-sectional studies that have shown a risk of incontinence after hysterectomy. However, in none of these studies were preoperative symptoms of incontinence recorded [13, 18, 24]. In the present study we showed that women with a normal-sized uterus (mean 5.3 GW) might have an increased risk of incontinence, while women with a larger uterus (mean 13.4 GW) greatly benefited from hysterectomy. Not only were they free of bleeding disorders, but urgency and leakage also decreased substantially. A prospective observational study based on patients’ self-reports after hysterectomy has also shown a decrease in stress urinary incontinence at both 1 year and 3 years [17]. Stratification according to size was not performed in that study. It has been argued that there is a time delay between acute trauma to the pelvic floor at childbirth and onset of symptoms such as urinary incontinence [28]. One can speculate that younger women are able to compensate for trauma to the pelvic floor to a greater extent than older women, so that the time to onset of symptoms after acute trauma would be shorter in older women. The possible influence of the time between hysterectomy and the onset of LUTS needs further evaluation.

Another limitation was the simple nature of the LUTS questions used during the period studied. We were not able to learn if the women who had reported both urinary incontinence and urinary urgency had leaks because of stress or urge incontinence because the original GynOp questionnaire was not designed to determine this. In 2006 a validated instrument with extended questions for LUTS quantification was introduced to the GynOp register [29]. We will soon have data to report from this cohort. Another weakness was the discrepancy in sample size between the two groups of women with similar uterine size: those with hysterectomy (group 1, 1,634 women) and those with hysteroscopy (group 3, 238 women). Hysteroscopic surgery is less common than hysterectomy for bleeding disorders [14], and we had to exclude 30% of otherwise eligible patients who had had a hysteroscopic procedure because uterine size data were missing. In spite of this, a significant difference was found between these groups in de novo incontinence after surgery, a finding well in line with results from epidemiological studies.

### Interpretation

This patient self-reported large register study with prospective data showed that evaluation of the risk of incontinence after hysterectomy in an individual patient needs to take into account uterine size and the presence of LUTS before surgery. A large Swedish epidemiological study with a mean follow-up time of 12 years has shown that hysterectomy increases the risk of subsequent surgery for stress urinary incontinence with a hazard ratio of 2.4 [18]. However, it did not include data on preoperative incontinence. The authors of that study concluded that the most plausible explanation for the increased hazard ratio was the effect of the surgical trauma inflicted during hysterectomy. The fact that several studies have failed to show differences in LUTS between patients with total and subtotal hysterectomy reduces the plausibility of the explanation [16, 23]. Moreover, neuroanatomical studies have shown that a simple hysterectomy is not likely to injure important neuroanatomical structures, e.g. bladder innervation [30]. Other studies have compared the outcomes after hysterectomy and hysteroscopy for LUTS. One randomized controlled study showed no differences in LUTS up to 2 years after surgery [26]. A retrospective study with a mean follow-up between 6 and 10 years with no preoperative data showed an increased risk of incontinence surgery amongst the women who had a hysterectomy than among those who had a hysteroscopy for heavy menstrual bleeding [14]. None of the above studies investigated the association between the size of the uterus and the risk of LUTS.

### Conclusion

The present study used data from a large existing register, the GynOp register, to study the risk of LUTS before and after hysterectomy in comparison with that after hysteroscopic procedures. LUTS was found to improve after hysterectomy if the symptoms had been present before surgery especially in women with a large uterus. De novo LUTS was also found to occur after hysterectomy in women with a normal-sized uterus.
